# Toxins induce ‘malaise’ behaviour in the honeybee (*Apis mellifera*)

**DOI:** 10.1007/s00359-014-0932-0

**Published:** 2014-08-23

**Authors:** Victoria Hurst, Philip C. Stevenson, Geraldine A. Wright

**Affiliations:** 1 Centre for Behaviour and Evolution, Institute of Neuroscience, Newcastle University, Newcastle upon Tyne, NE1 7RU UK; 2 Jodrell Laboratory, Royal Botanic Gardens, Kew, Surrey, TW9 3AB UK; 3 Natural Resources Institute, University of Greenwich, Chatham, Kent, ME4 4TB UK

**Keywords:** Honeybee, *Apis mellifera*, Malaise, Toxin, Aversion

## Abstract

To avoid poisoning and death when toxins are ingested, the body responds with a suite of physiological detoxification mechanisms accompanied by behaviours that in mammals often include vomiting, nausea, and lethargy. Few studies have characterised whether insects exhibit characteristic ‘malaise-like’ behaviours in response to intoxication. Here, we used the honeybee to investigate how intoxication produced by injection or ingestion with three toxins with different pharmacological modes of action quinine, amygdalin, and lithium chloride affected behaviour. We found that toxin-induced changes in behaviour were best characterised by more time spent grooming. Bees also had difficulty performing the righting reflex and exhibited specific toxin-induced behaviours such as abdomen dragging and curling up. The expression of these behaviours also depended on whether a toxin had been injected or ingested. When toxins were ingested, they were least 10 times less concentrated in the haemolymph than in the ingested food, suggesting that their absorption through the gut is strongly regulated. Our data show that bees exhibit changes in behaviour that are characteristic of ‘malaise’ and suggest that physiological signalling of toxicosis is accomplished by multiple post-ingestive pathways in animals.

**Electronic supplementary material:**

The online version of this article (doi:10.1007/s00359-014-0932-0) contains supplementary material, which is available to authorized users.

## Introduction

Eating exposes animals to the risk of ingesting toxic compounds. In response to toxicosis, the body mobilizes physiological detoxification mechanisms such as P450 enzymes and glutathione transferases used to break down toxic molecules for excretion (Jakobi and Ziegler 1990; Ioannides 2013). When mammalian subjects consume or are injected with toxins, their behaviour often changes. They spend less time moving, feeding and grooming and more time huddling and sleeping (Hart 1988; Millman 2007). Human subjects also report generalised pain or nausea. This suite of behaviours (aka ‘malaise’) can be caused by toxins (Nachman and Ashe 1973), by radiation (Garcia et al. 1955) and is also caused by infection (Dantzer and Kelley 2007).

Whether invertebrates also exhibit characteristic ‘malaise-like’ symptoms in response to toxicosis is poorly understood. Like mammals, invertebrates can learn to avoid cues associated with the consumption of toxins (Dethier 1980; Lee and Bernays 1990; Raffa 1987; Simoes et al. 2012). Furthermore, injection with lithium chloride (LiCl) (the canonical toxin used to produce malaise in vertebrates) causes crayfish to experience limb trembling, uncontrolled movements and periods of immobility (Arzuffi et al. 2000), and locusts injected with nicotine hydrogen tartrate are more likely to vomit (Simoes et al. 2012). Establishing whether there are behavioural reactions from intoxication that could be termed ‘malaise’ in invertebrates would pave the way for understanding the physiological mechanisms that produce these behaviours and for establishing whether they have an adaptive basis.

Honeybees are important model organisms for studying the neural basis of behaviour. Bees, like other animals, encounter toxins in their food (nectar and pollen) including pesticides that could potentially make them sick (Holzinger et al. 1992; Adler 2000; Alder et al. 2001; London-Shafir et al. 2003). We have recently established that honeybees have the ability to learn to avoid food cues associated with both the pre-ingestive and the post-ingestive consequences of encountering toxins (Wright et al. 2010). Bees will reject some toxins when they taste them, such as quinine, but appear to be unable to readily detect others like the almond nectar toxin, amygdalin, when such toxins are present in sucrose solutions (Wright et al. 2010). When bees inadvertently ingest amygdalin during associative learning, they learn to avoid odours associated with amygdalin-laced solutions using a post-ingestive signalling mechanism. It remains unclear whether toxins elicit behaviour in invertebrates that could be described as general ‘malaise’ as they do in mammals, or whether conditioned food avoidances arise from a separate mechanism.

Here, we tested whether ingestion of or injection with toxins results in a change in the behaviour of honeybees that could be termed ‘malaise.’ In this assay, we made continuous recordings of the locomotion and grooming of individuals within an hour after they experienced toxicosis. We used three toxins with the aim of identifying whether there were changes in behaviour common to toxicosis. The behaviours produced by injection were compared to those produced by ingestion.

## Methods

### Subjects

Adult foraging worker honeybees (*Apis mellifera*) were collected from an outdoor colony at Newcastle University during the summer; both nectar and pollen foragers were collected. Bees were also collected an indoor colony during winter; these bees were captured outside of the colony within the enclosed room. After collection, the bees were subjected to cooling anaesthesia and harnessed using standardised techniques (Bitterman et al. 1983). Once harnessed each bee was fed to satiation with 1.0 M sucrose and kept at room temperature overnight prior to experimentation.

### Treatments

Observations began 18–24 h after harnessing. The aim of the first experiment was to determine whether malaise response was observed in animals that had been injected with toxins. At 1 h before observation, 5 µl of 1.0 M sucrose was fed to each bee. Bees were cold anaesthetised 3 min prior to injection and injected subcuticularly in the thorax with 1 µl of the treatment solution using a 10 µl Hamilton syringe. Injection treatments were: water (the vehicle—referred to as ‘control’ in analysis), or 1 or 10 mM amygdalin; 0.1 or 1 mM quinine; 0.1 or 1 mM LiCl. All toxins were dissolved in deionised water; water was chosen instead of saline to improve solubility of the toxin. The aim of the second experiment was to determine whether bees exhibited a malaise response to ingestion of these toxins. At 1 h prior to the observation, each bee was fed 5 µl of a 1.0 M sucrose solution containing the toxin. Treatments were: the control (1.0 M sucrose) or a solution containing 1.0 M sucrose with 1 or 10 mM amygdalin; 0.1, or 1 mM quinine; 0.1 or 1 mM LiCl. [Note: the concentrations of the toxins were not the same because we had difficulty feeding bees with the higher concentrations of quinine, as in Wright et al. (2010)].

### Behavioural observations

Using an assay for locomotion in honeybees (Maze et al. 2006), we scored the following behaviours: walking, standing still, grooming, upside down, curled up, abdomen dragging and fanning and flying. In a pilot study, we observed that bees exhibited an unusual behaviour where they dragged their abdomens across the surface of the arena after consuming toxins. For this reason, we scored locomotion as two behavioural variables: walking normally (walking) and walking while the abdomen was dragging (abdomen dragging; Table 1). Additionally, we observed and scored three types of grooming behaviour during our experiments: proboscis grooming, body grooming and antennal grooming; these behaviours were pooled for the overall analysis because proboscis grooming and antennal grooming were each observed rarely (on average <2 % of total time budget). Observational arenas were composed of 100 mm × 15 mm plastic Petri dishes. After a 45-min period following treatment solution ingestion or immediately after injection, the subject was placed in the Petri dish and allowed to acclimate for 15 min before the observation began. Observations of 15 min periods were recorded live using the Observer software (Version 5, Noldus Information Technology).

**Table 1:** Definitions of recorded behavioural categories

Behaviour	Description
Walking	Walking and not displaying any other behaviour
Abdomen dragging	Walking and dragging back legs and abdomen on the floor of the arena
Stopped	Standing still
Upside down	On ventral surface and attempting to perform righting reflex
Curled up	Laying on its side and hunched up
Grooming	Rubbing antennae, body or proboscis with legs
Fanning/flying	Vigorously beating wings or in flight in arena

### Quantification of toxins in bee haemolymph

Bee haemolymph samples were obtained from individual honeybees fed 5 µl of one of the three doses of quinine (0.1, 1, or 10 mM) or amygdalin (1, 10, 100 mM) in 1.0 M sucrose. (Note: as above, the concentrations of quinine used were lower than for amygdalin because honeybees refused to consume solutions containing ≥10 mM quinine.) Each bee was cold euthanised, the abdomen removed, and haemolymph was extracted via centrifugation using the method described in Mayack and Naug (2010). Sample volumes were measured using 5 µl capillary tubes, and samples from individual bees fed the same toxin treatment were pooled to form 10 µl samples to which 10 µl of 50:50 methanol:water was added before the samples were frozen at −20 °C. Each sample was analysed for amygdalin or quinine using LC–MS using a Waters Alliance LC solvent delivery system with a ZQ MS detector on a Phenomenex Luna C18(2) column (150 × 4.0 mm i.d., 5 μm particle size) operating under gradient elution conditions, with *A* = MeOH, *B* = H_2_O, *C* = 1 % HCO_2_H in MeCN; *A* = 0 %, *B* = 90 % at *t* = 0 min; *A* = 90 %, *B* = 0 % at *t* = 20 min; *A* = 90 %, *B* = 0 % at *t* = 30 min; *A* = 0 %, *B* = 90 % at *t* = 31 min; column temperature 30 °C and flow rate of 0.5 ml min^−1^ for amygdalin and *A* = MeCN, *B* = H_2_O, *C* = 1 % HCO_2_H in MeCN; *A* = 0 %, *B* = 90 % at *t* = 0 min; *A* = 90 %, *B* = 0 % at *t* = 20 min; *A* = 90 %, *B* = 0 % at *t* = 30 min; *A* = 0 %, *B* = 90 % at *t* = 31 min; column temperature 30 °C and flow rate of 0.5 ml min^−1^ for quinine. Prior to LC–MS analysis, 60 µl of HPLC grade water was added to each sample and centrifuged at 12,000 rpm for 5 min; the supernatant was used for analysis. Amygdalin eluted at 5.91 min while quinine eluted at 5.30 min. Polynomial calibration curves for each compound via quantification of the [M + H]^+^ molecular ion of commercial standards (Sigma-Aldrich, Dorset, UK) in positive mode with *m/z* = 475.3 (amygdalin) and 325.3 (quinine) were used to quantify the concentrations of each compound in the haemolymph.

### Statistical analysis

Analysis of the percentage of the interval was performed using IBM SPSS software v19.0. The behavioural variables recorded in this analysis were mutually exclusive; therefore, their expression was correlated. To reduce the dimensionality of the data, factor analysis was performed using the principal components method of factor extraction with a Varimax rotation to increase data fit. The factor scores generated from the factor analysis were entered into a multivariate analysis of variance (MANOVA) to analyse the effect of toxins and route of administration on the performance of the behaviours; the scores represented the correlated behavioural variables and reduced the dimensionality of the data. Pairwise post hoc comparisons were made using a Dunnett’s post hoc test (Dunnett’s) performed against the control group only. For the analysis of concentration, the control group was not included in the MANOVA because separate control groups were not performed for each toxin. Comparisons of haemolymph toxins and behaviours that made up a small portion of the time budget (e.g. proboscis grooming) were carried out using a generalised linear model (GLZM) with Sidak’s post hoc comparisons (Sidak).

## Results

### Characteristics of toxin-induced malaise in bees

**Fig. 1:**
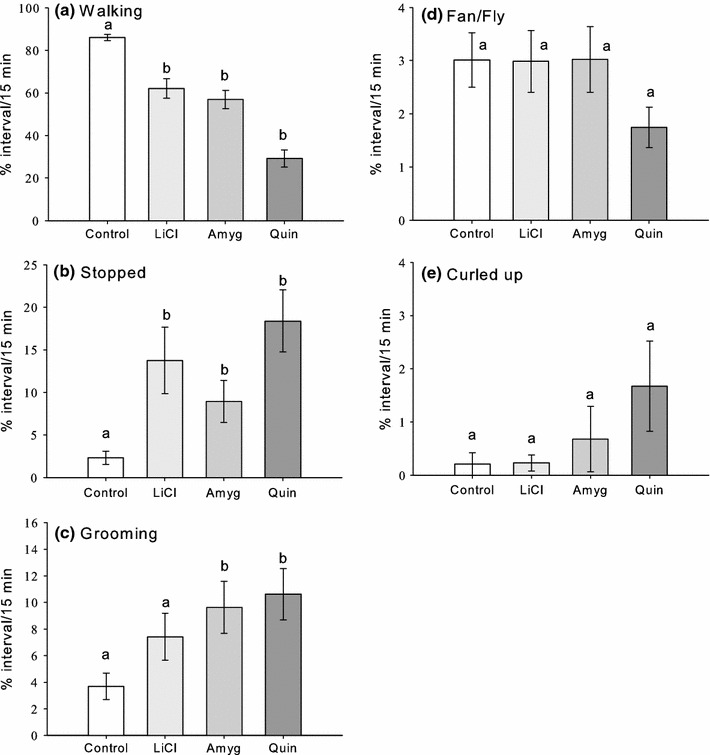
Toxicosis reduced the time spent walking, fanning, and flying and increased the time spent sitting still and grooming. Bees injected with or that ingested lithium chloride (LiCl), amygdalin (Amyg), or quinine (Quin) exhibited less walking (**a**), more time spent stopped (**b**), more time spent grooming (**c**), and less time fanning or flying (**d**). They were also more likely to exhibit curled up behaviour (**e**). *Letters* indicate Dunnett’s post hoc comparisons with control (*a*_1_ = injected bees, *a*_2_ = bees that ingested toxins); *differences in letters* indicate significance (*P* < 0.05). *Error bars* represent SE of the mean, *N*_control_ = 54, *N*_amgy_ = 58, *N*_LiCl_ = 51, *N*_Quin_ = 61

Bees spent most of their time walking during the assay (Fig. 1). Factor analysis revealed the correlations in the behaviours we recorded: time spent walking was positively correlated with fanning/flying and was negatively correlated with time spent stopped and grooming (Table 2, Factor 1). Two other behaviours, time spent upside down and time spent dragging the abdomen while walking, were also strongly positively correlated (Table 2, Factor 2). Time spent curled up was not strongly correlated with the other behavioural variables (Table 2, Factor 3).

**Table 2:** Factor analysis of toxin-induced behaviours

	Factor		
	1	2	3
Eigenvalue	1.9	1.5	1.2
% Variance explained	27.9 %	21.6 %	17.3 %
Walking	**−0.758**	−0.479	−0.306
Stopped	**0.851**	−0.245	−0.114
Grooming	**0.584**	−0.359	0.419
Fanning/flying	**−0.560**	−0.280	0.018
Upside down	−0.004	**0.741**	−0.228
Dragging abdomen	0.065	**0.686**	0.365
Curled up	0.006	0.022	**0.865**

Factor analysis of all data. Fit accomplished using a Varimax rotation. Coefficients for variables with strong contributions (>0.5) are in bold

If bees had been injected with or had ingested toxins, they spent less time walking, fanning/flying and more time stopped and grooming (Fig. 1a–d; Table 3, MANOVA, toxin main effect, *F*_3,216_ = 11.1, *P* < 0.001). Injection and ingestion of toxins affected these behaviours in a similar way (Table 3, MANOVA, route of administration main effect, *F*_1,216_ = 0.028, *P* = 0.867). Bees experiencing toxicosis also spent more time curled up (Fig. 1e; Table 3, MANOVA, toxin main effect, *F*_3,216_ = 5.32, *P* < 0.001) and this was true whether they had been injected with toxins or had ingested them (Table 3, MANOVA, route of administration main effect, *F*_1,216_ = 1.68, *P* = 0.196). Overall, curled up behaviour was seen less than 3 % of the time and was specific to intoxication.

**Table 3:** MANOVA of the factor scores for the factor analysis in Table 2

Model term	*F* (*df*)	*P* value
Toxin		
Factor 1	11.1 (3,216)	<0.001
Factor 2	10.7 (3,216)	<0.001
Factor 3	5.32 (3, 216)	0.001
Route of admin		
Factor 1	0.028 (1,216)	0.867
Factor 2	1.24 (1,216)	0.266
Factor 3	1.68 (1,216)	0.196
Toxin × route of admin		
Factor 1	0.189 (3,216)	0.904
Factor 2	7.67 (3,216)	<0.001
Factor 3	0.486 (3,216)	0.692

### The righting reflex and abdomen dragging behaviour reveal toxic action

**Fig. 2:**
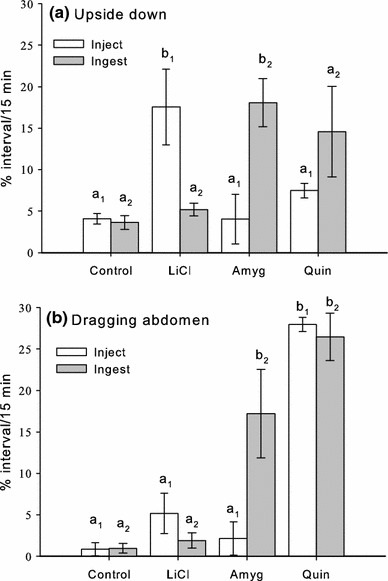
Failure of the righting reflex and abdomen dragging reflect acute malaise caused by injection or ingestion of toxins. **a** Failure of the righting reflex (*upside down*) depended on whether the toxin had been injected or ingested and the type of toxin administered. *N*_control_ = 24, *N*_amgy_ = 29, *N*_LiCl_ = 21, *N*_Quin_ = 31. **b** Abdomen dragging behaviour was greatest in bees injected with or that had ingested quinine. *Letters* indicate Dunnett’s post hoc comparisons with control (*a*_1_ = injected bees, *a*_2_ = bees that ingested toxins); *differences in letters* indicate significance (*P* < 0.05). *Error bars* represent SE of the mean. *N*_control_ = 30, *N*_amgy_ = 29, *N*_LiCl_ = 30, *N*_Quin_ = 30

Two variables that were strongly influenced by toxin ingestion or injection were the amount of time spent upside down (the failure to perform the righting reflex) and the amount of time spent dragging the abdomen (Fig. 2). Upside down behaviour was as much as 20 % of the entire interval in some cases of toxicosis, but was never more than 5 % of the interval in control bees (Fig. 2a). Abdomen dragging behaviour was largely peculiar to bees that had ingested toxins (Fig. 2b).

Whether or not a given toxin influenced either of these behaviours, however, depended on if it was injected or ingested by the bees (Table 3, MANOVA, toxin × route of administration, *F*_3,216_ = 7.67, *P* < 0.001). The effect of LiCl on these two behaviours, for example, depended on how it was administered. Injection with LiCl was more likely to cause a failure to right (Dunnett’s post hoc, *P* = 0.001) whereas ingestion did not (Dunnett’s post hoc, *P* = 0.980). Neither injection (Dunnett’s post hoc, *P* = 0.737) nor ingestion of LiCl (Dunnett’s post hoc, *P* = 0.996) affected abdomen dragging. In contrast, the toxic action of amygdalin depended on whether it had been ingested. Bees that had ingested amygdalin spent up to 20 % of their time upside down (Dunnett’s post hoc, *P* = 0.015), but were not different to the control when they had been injected with these toxins (Dunnett’s post hoc, *P* = 1.0). They also spent more time dragging the abdomen when they had ingested amygdalin (Dunnett’s post hoc, *P* = 0.008) but did not exhibit this behaviour more often than the control when it had been injected (Dunnett’s post hoc, *P* = 0.986). Quinine caused a (marginally) higher probability of time spent upside down when ingested (Dunnett’s post hoc, *P* = 0.081), but not when injected (Dunnett’s post hoc, *P* = 0.637). It also elevated time spent dragging the abdomen to over 25 % of the interval in both conditions (Dunnett’s post hoc, both *P* < 0.001).

When grooming was being scored during observations, it was split into three behaviours: proboscis grooming, body grooming, and abdomen grooming. In a separate analysis, we also found that each of these behaviours reflected whether a toxin had been injected or ingested (proboscis: GLZM, toxin × route of administration, *χ*_3_^2^ = 17.6, *P* = 0.001). For example, quinine, a toxin that has previously reported to taste bitter to bees, caused an elevation of proboscis grooming (relative to the control) after it had been ingested (and had been in contact with the mouthparts) but not when it was injected (Sidak, *P* < 0.001); LiCl (Sidak, *P* = 0.999) and amygdalin (Sidak, *P* = 1.0) had no effect on proboscis grooming. Body grooming and abdomen grooming, on the other hand, were not affected by toxin type (body: GLZM, *χ*_3_^2^ = 4.98, *P* = 0.173; abdomen: GLZM, *χ*_3_^2^ = 2.22, *P* = 0.527) or route of administration (body: GLZM, *χ*_1_^2^ = 1.64, *P* = 0.200; abdomen: GLZM, *χ*_1_^2^ = 1.30, *P* = 0.253).

### Effect of concentration on the expression of acute malaise

**Fig. 3:**
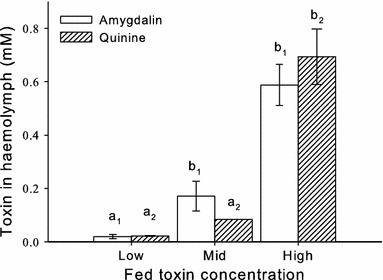
Amount of toxin fed to bees was > tenfold lower than that recovered in haemolymph. Bees were fed amygdalin (low = 1 mM, mid = 10 mM, high = 100 mM) or quinine (low = 0.1 mM, mid = 1 mM, high = 10 mM) at 1 h prior to haemolymph sampling. *Letters* indicate Sidak’s post hoc comparisons with control (*a*_1_ = amygdalin, *a*_2_ = quinine); *differences in letters* indicate significance (*P* < 0.05). Values are means of pooled samples, *error bars* represent SE of the mean. *N*_low_ = 4, *N*_mid_ = 3, *N*_high_ = 4

Injection of toxins provides a controlled way of delivering toxins in laboratory conditions; because toxins are almost always acquired by ingestion, injection does not reflect how most animals experience toxins. To identify how much of the ingested toxins passed over the gut, we measured the toxins amygdalin and quinine in the haemolymph of honeybees after feeding them a specific dose (Fig. 3). Bees fed the highest concentration had more toxin in the haemolymph (GLZM, concentration main effect, *χ*_2_^2^ = 237, *P* < 0.001). When fed 10 mM (high) quinine or 100 mM (high) amygdalin, bees had an almost tenfold lower concentration in haemolymph than the fed dose.

To verify that the toxins injected into the bees or ingested by the bees were the cause of the change in behaviour, we performed separate factor analyses on the two routes of administration for the concentrations of the toxins we tested (Figs S1 and S2). The concentration of the toxin in the range we tested (0.1–10 mM) did not have a significant influence on the expression of walking, stopped, grooming or fanning/flying behaviour when injected (Fig S1, Table S1, MANOVA, concentration main effect, *F*_1,75_ = 0.260, *P* = 0.111) or ingested (Fig S1, Table S2, MANOVA, concentration main effect, *F*_1,112_ = 0.404, *P* = 0.526). We also tested whether toxin concentration influenced the expression of upside down, abdomen dragging, and curled up behaviour (Fig S2). When injected, whether or not the toxin caused these behaviours depended on both the toxin concentration and the type of toxin (Table S1, MANOVA, concentration × toxin, *F*_2,75_ = 4.99, *P* = 0.009). When ingested, however, the expression of these behaviours did not depend on toxin concentration (Table S2, MANOVA, concentration main effect, *F*_1,112_ = 0.404, *P* = 0.526).

## Discussion

Our data represent the first complete characterisation of behaviours caused by the feeding or injection of toxins in an invertebrate. When injected or ingested, all three toxins reduced time spent walking, increased the time spent still, and increased time spent grooming. Both injection and ingestion of toxins caused failure of the righting reflex and caused the expression of abnormal behaviour such as abdomen dragging or curling up that were rarely or never observed in the control subjects. Some toxins were more effective if injected and others when ingested; for example, LiCl had a stronger influence on behaviour when injected than when ingested, whereas amygdalin and quinine had stronger influences when ingested. We predict that both the gut and the central nervous system can respond to toxins directly and have a shared mechanism for signalling toxicosis that targets the control of motor function.

### Reduced locomotion is a hallmark of malaise

Our data agree with previous work in rats (Johnson 1979; Wolthuis et al. 1975; Cappeliez and White 1981) and clearly show that a key characteristic of the change in state caused by toxicosis in animals is an immediate reduction in locomotion. The adult honeybees in the control group of our experiments were very active in our locomotion assay, spending over 80 % of their time walking during the 15 min observation period. Insult with toxins reduced this activity by as much as 45 % and was accompanied by an increase in time spent still. Spending less time walking could conserve metabolic resources used to neutralise toxicosis, as detoxification commands ATP and amino acids to mobilise the production of enzymes and active transport for the excretion of toxins (Cresswell et al. 1992; Lochmiller and Deerenberg 2000; Bains and Kennedy 2004). This idea is supported by the fact that Madagascar hissing cockroaches (*Gromphadorhina portentosa*) exposed to pesticides have a lower metabolic rate (Sawczyn et al. 2012), and energy reserves in the earthworms (*Enchytraeus albidus*) are depleted during a recovery from metal toxicosis (Novais et al. 2013). We predict that because metabolic resources are required for detoxification, the ingestion of toxins could be particularly harmful to foraging bees. Foragers require foods high in carbohydrates to produce enough ATP to fly [for review see Rothe and Nachtigall (1989), Harrison and Roberts (2000)]. If they are forced to use carbohydrates and amino acids to detoxify ingested toxins, they are likely to face a trade-off between detoxification and foraging for the colony that depends on how much ATP is required for detoxification. In addition, bees and other animals may avoid dangers posed by predators or other hazards by remaining still while recovering from toxicosis (Hart 1988; Aubert 1999), if detoxification commands physiological resources required to elicit the appropriate escape response.

### Malaise state is indicated by a failure to right, more time grooming, and performance of toxin-specific behaviours

Bees fed or injected with toxins also exhibited toxin-specific behaviours such as assuming a curled up posture. It is unclear what this behaviour represents, but insects often die in this position. Interestingly, we observed these behaviours in animals that had been injected with toxins as well as those that ingested them. In vertebrates, curling up behaviour has also been described as a hallmark of sickness that might have the adaptive value of conserving body heat (Hart 1988). Another peculiar behaviour we observed was ‘abdomen dragging’, and this only occurred in animals that had been treated with toxins. Rats injected with LiCl display ‘body dragging’ where the body is elongated and the belly dragged along the floor by the front paws, writhing [a concavity in the flank of the animal caused by muscular contractions, Parker (1982), Parker et al. (1984) and Ohmura et al. (2012)], or ‘lying on belly’ (Parker et al. 1984; Parker 1982; Meachum and Bernstein 1990). This behaviour in rats, in particular, is characterised by a flattened torso, limp limbs and laying the head down and has been previously interpreted to indicate that rats feel pain associated with toxicosis (Meachum and Bernstein 1990). It is also observed in response to procedures expected to cause abdominal pain (Roughan and Flecknell 2003). It is interesting to note that we observed this behaviour in bees that had been injected with the toxins as well as those that had ingested quinine, suggesting that the activation of this behaviour is independent of the toxins passing over the gut.

Bees spent more time grooming in all toxin treatment conditions. For this reason, we predict that it is one of the key characteristics that defines toxin-induced malaise in insects. Interestingly, ingestion of pesticides and pharmacological agents like ethanol in food also elevates the time that bees spend grooming (Neuman-Lee et al. 2013; Williamson et al. 2013). In contrast, vertebrate animals often stop or reduce grooming in response to toxicosis or pathogen-induced illness (Ritter and Epstein 1974; Parker et al. 1984; Hart 1988; Meachum and Bernstein 1990; Kulikov et al. 2010; Tikhonova et al. 2011; Bassi et al. 2012). Thus, time spent grooming is a clear difference in ‘malaise’ behaviours between mammals and insects. In insects, self-grooming is a means of removing external parasites (Boucias and Pendland 1998; Rath 1999; Currie and Tahmasbi 2008), and antennal and mouthparts grooming enhances the senses of taste and smell (Jacquet et al. 2012). Grooming could be an adaptive trait if it increased the detection and elimination of their parasites.

Our data also showed that toxins influenced the expression of the righting reflex and abdomen dragging. The expression of these behaviours, however, depended on whether a toxin had been ingested or injected. We propose that the expression of these two behaviours indicates an acute state of toxicosis in insects. In our study, LiCl did not significantly affect these behaviours when it was ingested, perhaps indicating that its uptake into the haemolymph, like that of salts in other insects, is strongly restricted by the gut (Trumper and Simpson 1993). In contrast, amygdalin was more likely to cause time spent upside down and abdomen dragging when ingested but not injected. Amygdalin may not be as toxic when injected because its mode of action depends on contact with beta-glucosidase enzymes mainly present in the gut and crop that break it down into cyanide (Conn 1969; Pontoh and Low 2002). Quinine, on the other hand, produced abdomen dragging and upside down behaviour whether it had been injected or ingested. Quinine blocks sodium channels, and these channels are present in the gut and also in nerve and muscle cells throughout the body, so its targets are not restricted to the gut.

### Toxins are sensed by the gut and other organs

In general, our data show that toxin-induced ‘malaise’ in insects is characterised by more time spent grooming and more time spent performing of specific behaviours such as being unable to perform the righting reflex, being curled up, or abdomen dragging. However, there were subtle differences in expression that depended both on the toxin and the way it was administered. When injected directly into the haemolymph, a toxin gains direct contact with tissues and organs within an animal. Our data show that ingestion also results in toxins being delivered to the haemolymph and, therefore, also to the other tissues. Our data indicate that both the gut and organs in contact with the haemolymph respond to the presence of toxins in a way that alters behaviour.

While the gut is an important barrier to prevent toxicosis by actively inhibiting transport of toxins in the haemolymph, it also houses cells that express detoxifying enzymes such as p450 enzymes. It is likely that any toxin that was ingested would be attacked by these enzymes in the gut and reduce the toxin load. Thus, detection of toxins and signalling by the gut could be one of the first forms of a physiological ‘malaise’ response that could also influence behaviour perhaps via peptidergic signalling by enteroendocrine cells in the gut (Chen et al. 2006; Glendinning et al. 2008). This would explain why the concentrations we found in the haemolymph were lower than those fed to the bees.

However, our data also show that toxins cross the bee’s gut and are found in the haemolymph. Once in the haemolymph, they would be free to interact with the brain or other organs prior to detoxification by p450 enzymes in the Malpighian tubules and subsequent excretion (Yang et al. 2007). Recent expression studies in insects have shown that gustatory receptors are expressed in non-canonical locations in insects including the gut (Park and Kwon 2011) and the brain (Thorne and Amrein 2008; Miyamoto et al. 2012, 2013); such receptors could act as sensors to mobilise physiological defences and alter behaviour when toxins were present in haemolymph. Based on studies of other toxic or pharmacologically active substances such as caffeine ingested by bees, we expect that toxins can cross the blood–brain barrier to act directly on circuits that regulate behaviour (Mustard et al. 2012; Wright et al. 2010, 2013). This idea is supported by the fact that two studies of associative olfactory conditioning in honeybees have identified that bees previously fed solutions containing quinine or amygdalin are less likely to extend their proboscis towards odours predicting reward and less likely to feed (Ayestaran et al. 2010; Wright et al. 2010). Likewise, locusts that have been injected with the toxin, nicotine hydrogen tartrate, also learn to avoid odours associated with the consequences of toxin injection (Simoes et al. 2012). Identification of the extent to which these toxins directly act on the nervous system, and whether there are specific mechanisms for directly detecting toxins in the brain or in other ganglia or organs will be the subject of future investigations.

Based on our measurements of toxins in the haemolymph after the consumption, we suggest that ingestion could potentially lead to a slower rate of toxin dose administration than injection because bees can regulate the rate of passage of the food from crop to midgut (Blatt and Roces 2001). Post-ingestive feedback mechanisms that detect toxins in food exist in the insect crop and the gut (Park and Kwon 2011). For example, gustatory receptors in enteroendocrine cells in the gut (Park and Kwon 2011) may mediate nutrient absorption (Miguel-Aliaga 2012; Miyamoto et al. 2013) and could also detect toxins. These cells also signal the presence of nutrients and toxins to other tissues via peptidergic signals including cytokinins (Behrens and Meyerhof 2011). Such signals are likely to be the primary means by which the gut signals a state of toxicosis to the rest of the body. We predict that receptors for toxicosis-induced peptides or other chemical signals are also present in regions of the insect brain (e.g. the suboesophageal ganglion) that facilitate the change in motor function that drive changes in behaviour that characterise malaise. These receptors could also exist in nerve chord ganglia.

Our study is the first to characterise the change in behaviour caused by toxin consumption and injection in the same organism. All three toxins each had different pharmacological targets but still produced a similar suite of behaviours in bees. A previous study in honeybees using these same three substances also found that bees were less likely to consume food after they had ingested these toxins (Ayestaran et al. 2010), supporting the idea that bees experience a generalised ‘malaise’ after consuming toxins that alters behaviour. The physiological pathways associated with the production of malaise are unknown in any animal, but like studies using LiCl in rodents, our data show that it is possible to produce malaise without toxins interacting directly with the gut. That some of these behaviours such as the reduction in locomotion and an increase in malaise-specific behaviours are common to rats and bees implies that malaise is an evolved adaptation that increases survival.

## Electronic supplementary material

Below is the link to the electronic supplementary material.

Supplementary material 1 (EPS 34629 kb)

Supplementary material 2 (EPS 34508 kb)

Supplementary material 3 (DOCX 17 kb)
