# Supplementary material for: Toxins induce ‘malaise’ behaviour in the honeybee (Apis mellifera)
Source: J Comp Physiol A Neuroethol Sens Neural Behav Physiol. 2014 Aug 23;200(10):881–90. doi: 10.1007/s00359-014-0932-0 (PMC4169619; doi:10.1007/s00359-014-0932-0)
Supplement: Supplementary file 3 — Supplementary material 3 (DOCX 17 kb) [file 359_2014_932_MOESM3_ESM.docx]

**Supplementary material**

**Figure S1**. Effect of concentration of ingested or injected toxins on the expression of motor function in bees (behaviours associated with Factor 1 in Table S1 and S2). A = amygdalin, L = LiCl, and Q = quinine. Numbers on the x-axis refer to the concentration that was injected or ingested by the bees.

**Figure S2**. Effect of concentration of ingested or injected toxins on the expression of malaise related behaviours in bees (behaviours associated with Factor 2 in Table S1 and S2). A = amygdalin, L = LiCl, and Q = quinine. Numbers on the x-axis refer to the concentration that was injected or ingested by the bees.

**Table S1**

|  | **factor** | |
| --- | --- | --- |
|  | 1 | 2 |
| Eigenvalue | 2.1 | 1.5 |
| % variance explained | 30.4% | 27.7% |
| Walking | **-0.755** | **-0.552** |
| Stopped | **0.81** | -0.174 |
| Grooming | **0.72** | -0.183 |
| Fanning/Flying | **-0.539** | -0.258 |
| Upside Down | -0.252 | **0.551** |
| Dragging Abdomen | 0.167 | **0.803** |
| Curled Up | 0.038 | **0.747** |

(Factor analysis of injection data. Fit accomplished using a Varimax rotation. Coefficients for variables with strong contributions (>0.5) are in bold.)

**Table S2**

|  | **factor** | | |
| --- | --- | --- | --- |
|  | 1 | 2 | 3 |
| Eigenvalue | 2.1 | 1.5 | 1.1 |
| % variance explained | 30.7% | 21.4% | 15.6% |
| Walking | **-0.898** | 0.283 | -0.002 |
| Stopped | **0.631** | 0.421 | -0.486 |
| Grooming | **0.613** | 0.513 | 0.216 |
| Fanning/Flying | **-0.543** | 0.212 | 0.339 |
| Upside Down | 0.125 | **-0.73** | -0.31 |
| Dragging Abdomen | 0.287 | **-0.619** | 0.465 |
| Curled Up | 0.425 | 0.136 | **0.619** |

(Factor analysis of ingestion data. Fit was not subject to rotation. Coefficients for variables with strong contributions (>0.5) are in bold.)
